# The Impact of Neural Stem Cell Biology on CNS Carcinogenesis and Tumor Types

**DOI:** 10.4061/2011/685271

**Published:** 2011-05-19

**Authors:** K. M. Kurian

**Affiliations:** Department of Neuropathology, Frenchay Hospital, Bristol BS16 1LE, UK

## Abstract

The incidence of gliomas is on the increase, according to epidemiological data. This increase is a conundrum because the brain is in a privileged protected site behind the blood-brain barrier, and therefore partially buffered from environmental factors. In addition the brain also has a very low proliferative potential compared with other parts of the body. Recent advances in neural stem cell biology have impacted on our understanding of CNS carcinogenesis and tumor types. This article considers the cancer stem cell theory with regard to CNS cancers, whether CNS tumors arise from human neural stem cells and whether glioma stem cells can be reprogrammed.

## 1. Introduction

 Epidemiological data suggests that the incidence of gliomas—the most common form of intrinsic brain tumour—is rising [1–3]. This is surprising, because the brain is partially protected from the environment factors by the blood-brain barrier and has a low proliferative potential compared with other organs. Recent advances from the stem-cell biology field have impacted on our understanding of CNS carcinogenesis and tumor types [4–7]. This paper considers the evidence for the cancer stem cell theory, whether CNS tumours arise from human neural stem cells and whether glioma stem cells can be reprogrammed. 

## 2. Cancer Stem-Cell Theory

 Normal human neural stem cells are thought to reside within the brain mostly in the subventricular zone (SVZ) lining the lateral ventricles and within the dentate gyrus of the hippocampus [8, 9]. These stem cells persist throughout adulthood into old age and may divide symmetrically for self-renewal and asymmetrically to produce neurons, astrocytes, and oligodendrocytes [10]. By comparison, brain cancer or glioma stem cells are a population within a glioma that can divide infinitely, have the capacity to show neuronal, astrocytic, and oligodendroglial differentiation and can recapitulate the whole tumour when transplanted into the brain of a nude mouse [11–13]. Whether brain cancer stem cells actually develop from preexisting human neural stem cells or represent cells which reacquire a stem-like state as a by product of tumorigenesis or in vitro culture conditions remains controversial [11–13]. Brain cancer/glioma stem cells are of great interest, because they may represent the population of cells within a tumour that may be resistant to therapy and responsible for tumour relapse.

 Cancer stem cells, were originally described in acute myeloid leukaemia and in haematological malignancies and subsequently in many solid tumours [4, 6, 7]. Brain cancer stem cells, also termed brain tumour-initiating cells were first described by groups who used CD133 to isolate a population of brain tumour initiating or stem cells within glioblastoma, the most aggressive primary brain tumour [12, 14, 15]. The precise function of CD133, also known as prominin, remains unclear; however, it was originally shown to be a haemopoietic stem-cell marker [10]. Original papers suggested that CD133-positive stem-like cells were the only subpopulation of cells within the glioblastoma that were capable of producing tumours when transplanted into the brain of immunodeficient mice [12, 14]. More recent studies suggest that this may not always be the case and that CD133 is not a specific marker for brain tumour-initiating cells or cancer stem-cells within glioma [15]. Therefore many experimental difficulties still revolve around the lack of specific glioma stem cell markers.

 The question whether there is a universal glioma cancer stem cell or whether different subtypes of glioma contain different stem cells remains unanswered. Gilbertston's group suggest that the stem cell may vary according to the nature of the original tumour [16]. He has shown that subtypes of a different type of glial tumour (ependymoma) may derive from radial glia at different locations at different stages of development in the nervous system. It is possible that radial glia may represent the stem cell of ependymomas [17]. These cells express CD133, RC2, and nestin, which are present on radial glia and human neural stem cells. 

 In addition glioma stem cells are dependent on their microenvironment in order to maintain stem-cell properties. There is evidence to suggest that endothelial cells interact and secrete factors in vitro that maintain a stem-like state [17]. Increasing the number of endothelial cells expands the population of self-renewing cells and their tumorigenic properties. It is therefore possible that stem-like cells survive within a vascular niche [17]. 

## 3. Do CNS Tumours Arise from Human Neural Stem Cells?

 It is difficult to determine whether CNS tumours arise from human neural stem cells because of the difficulties targeting these cells and the lack of robust stem-cell markers. Recent work suggests that different combinations of genetic mutations in the adult mouse subventricular zone determine different brain tumour phenotypes [18, 19]. The SVZ is a well-described niche containing stem cells (type B cells), transient amplifying precursors which derive from them (type C cells), and neuroblasts (type A cells) [20, 21]. Many studies use a GFAP-cre-mediated approach in transgenic mice to target type B stem cells expressing GFAP and subsequent progenitors expressing Nestin during development. One intriguing study has shown that stimulation of the PDGFR alpha expressing B-type neural stem cells induces the formation of hyperplasia-resembling oligodendrogliomas next to the SVZ [22], which regresses after the withdrawal of the growth factor. Similar approaches have shown that the inactivation of various combinations of other tumour suppressor genes forms malignant astrocytomas in the developing mouse brain SVZ. The inactivation of Nf1 and p53 in neural stem/progenitor cells of the SVZ and the inactivation of PTEN and p53 in progenitor cells result in the formation of malignant astrocytomas [23, 24]. Other studies have shown that the activation of oncogenes Ras and Akt in nestin-expressing progenitors (but not in GFAP-expressing SVZ stem cells) induces glioblastoma [25]. Similarly, nestin-expressing GFAP-negative progenitor cells deficient in INK4a/ARF and Bmi1, isolated in vitro, can give rise to low-grade diffuse astrocytomas [26]. 

 Experiments in the adult SVZ have used a similar cre-lox method in transgenic mice. The inactivation of combinations of Nf1 and p53, or Nf1, p53 and PTEN, in Nestin expressing cells results in the formation of malignant gliomas [27, 28]. An interesting study combining the inactivation of Rb and p53 in GFAP-expressing cells of the adult subventricular zone resulted in the formation of primitive neuroectodermal tumours (PNET) rather than gliomas, suggesting that the inactivation of Rb may be key in determining this tumour phenotype [17]. 

 Although these experimental models are compelling evidence for the development of brain cancers from neural stem cells, there are several reports indicating that transformed mature astrocytes outside the SVZ can form gliomas given appropriate mutations [29–33]. This is coupled with the clinical observation of glioma occurrence in locations outside the traditional stem-cell compartments, and therefore the jury is still out on whether all CNS tumors arise from human neural stem cells. 

## 4. Can We Reprogramme Glioma Stem Cells?

A major conceptual advance in stem-cell biology field revolves around the work by Takahashi et al. [34]. Yanamaka “reprogrammed” mature skin fibroblasts into induced pluripotent stem cells, commonly abbreviated as iPS cells or iPSCs with four factors: Oct4, klf4, sox 2, and c-myc using a retroviral aproach [34]. These iPS cells are similar to natural pluripotent stem cells, such as embryonic stem cells because they have the potential to be differentiated into different cell types such as nerve or cardiac cells. This advance theoretically allows researchers to obtain pluripotent stem cells from skin or other mature tissues without the controversial use of embryos. It also avoids immune rejection because the cells could be derived entirely from the individual patient to be treated. 

More fundamentally, it demonstrates the influence of epigenetics in deciding the phenotype of an individual cell, and raises the possibility that epigenetics could be used to alter cell fate. Initial concerns about the risk of malignancy in reprogrammed human neural stem cells have revolved around the use of c-myc and viral transduction techniques. These have been addressed by using alternative techniques including the 2iIPS system [35], piggy bac transposons [36] and protein based approaches [37, 38]. In the CNS tumour field it has been shown both in vivo and in vitro that it is possible to make certain types of glioma-initiating stem cells differentiate into neuronal type cells using manipulation of the Bone Morphogenetic Protein (BMP) pathway, which is involved in differentiation of human embryonic stem cells to neural stem cells [39, 40]. These studies demonstrated a major differentiation block in a subset of glioblastoma is caused by the Polycomb repressor complex-(PRC-) mediated epigenetic silencing of the BMPR1B promoter analogous to early embryonic neural stem cells.

 Very recent work (Stricker et al., in press) has shown that direct reprogramming of the glioblastoma epigenome restores developmental potential but does not efficiently suppress proliferation [41]. This fundamental study uses a reprogramming approach to convert glioblastoma into immature teratoma.

## 5. Conclusions

 In conclusion, the cancer stem theory predicts that not all tumour cells are equal. Although we cannot be certain about the precise cell of origin of gliomas, we may have the ability to control them to a certain degree using epigenetic means. This represents a major conceptual advance in the field, but it is still contraversial. Major new insights into cancer stem cells will hopefully direct a new era of patient-specific combined molecular therapies.
